# Ptomaine Poisoning—III
*The preliminary articles of this Clinic appeared in The Hospital for August 19 and 26.


**Published:** 1911-09-09

**Authors:** H. J. Hutchens

**Affiliations:** Heath Professor of Comparative Pathology and Bacteriology in the University of Durham.


### Hospital Clinics.

### PTOMAINE POISONING—III.*

By H. J. HUTCHENS, D.S.O., D.P.H. Oxon., M.A.

Heath Professor of Comparative Pathology and Bacteriology in the University of Durham.

Another important result of Bainbridge's work has been to show that the various rat viruses (*B. Danysz*, *B. typhi murium*, etc.) are, bacteriologically, pure or mixed cultures of *B. Gärtner*, *B. Aertrycke*, or *B. paratyphoid β*. The organisms present in the different rat and mouse viruses, as well as certain other organisms, *e.g.* Nocard's psittacosis bacillus, had previously been regarded as closely related to Gärtner's bacillus, but their identity had not been proved. Objection has been raised to regarding the various rat and mouse viruses as identical with the food-poisoning bacilli on the ground that were they identical epidemics would have occurred among those who handled such viruses. This objection is easily met, because the handling of these viruses can be shown to have resulted in some instances in an outbreak of food-poisoning among those who handled them. In this connection the following observation of Shibayama in Japan (quoted by Sacquépée) is of interest: "In a village in the province of Tajamata a quantity of *B. typhi murium*, which was intended for use as a rat poison, was by mistake given to a horse which at the time was in perfectly sound health. The horse became ill the same day and died within a week. The carcase was buried but was subsequently dug up by a number of workmen, who ate the flesh. Within three days thirty-four of those who had eaten some of the horseflesh fell ill and one of them died. The organism was recovered from the meat."

The so-called bacillus of hog cholera is identical with *B. Aertrycke*. This organism appears to be a secondary infection in pigs suffering from hog cholera, the true cause of the disease being one of the invisible, filtrable viruses. The identity of *B. Aertrycke* and *B. suipestifer* does not appear to be disputed, and yet very large numbers of carcases of pigs which have died of hog cholera, many of them presumably being infected with *B. Aertrycke*, are ing is often associated with the consumption of the annually consumed as food, and though food-poison-meat of pigs, yet the number of such epidemics is very small as compared with what might have been expected.

2. Food-poisoning due to *B. botulinus* (Botulism) is a much rarer form of food-poisoning than that just considered. The symptoms are quite different from those produced by the enteritidis bacilli and chiefly affect the nervous system. The incubation period is short (12 to 24 hours) and is generally followed by some feeling of nausea, abdominal pain and constipation, but the characteristic symptoms develop later, resembling closely the symptoms due to poisoning by the vegetable alkaloids, dysphagia, dryness of the mouth, marked dilation of the pupil, paralysis of accommodation, ptosis, etc.

The disease generally lasts some weeks, but may be merely of a few hours' duration. The death-rate is high and varies from 15 to 40 per cent. of those attacked.

The symptoms of botulism are due to poisoning with the toxins of a spore-bearing bacillus, *Bacillus botulinus*, which was discovered by van Ermengen in 1895 in an epidemic of food-poisoning following a banquet at Ellezelles. The organism was isolated from the ham which gave rise to the illness, and also from the spleen and intestinal contents of one of the fatal cases. The *Bacillus botulinus* is a strict anaerobe and gives rise to an extra-cellular toxin chemically analogous to the toxins of diphtheria and tetanus. Botulism is always associated with the consumption of meat which has been preserved, and preserved under anaerobic conditions.

3. Symptoms of food-poisoning sometimes follow the consumption of meat infected with organisms other than those already referred to. The nature of the infection in these cases is not definitely ascertained but would seem to be toxæmia, but whether the substance producing the symptoms is a true bacterial toxin or not is difficult to determine. Of five epidemics of food-poisoning occurring in Newcastle and the neighbourhood during the second six months of last year, three belong to this category. All of them followed the use of tinned meat. In two cases corned beef was the cause and in one case tinned salmon.

On September 15, 1910, six persons partook of corned beef from a newly-opened 7-lb. tin. A few hours (2 to 6½) later they all fell ill and the symptoms were those of a severe gastro-enteritis with collapse. The meat was bacteriologically examined and the only organism recovered was the *Staphylococcus aureus*. The meat was wet, in parts of a greenish appearance, and had a disagreeble odour. All the patients recovered. Other tins of the same brand exposed for sale in the same shop gave rise to no untoward symptoms when eaten by other customers. Ostertag refers to an epidemic of food poisoning investigated by Kuborn at Denis which was considered to be due to the *Staphylococcus aureus*.

In the other epidemic of food-poisoning following the consumption of corned beef, at least nineteen people partook of the beef and all of them suffered from a more or less severe illness. The symptoms were preceded by a short incubation period (1½ to 7 hours) and were of the nature of an acute gastro-enteritis. A proteus was isolated from the meat and was probably the cause of the illness. No deaths occurred. A portion of the meat was examined in London on behalf of the agents for ptomaines, but I am informed that no substances of that nature were present.

The third epidemic concerned five persons, a man, his wife, and his three young children, all of whom ate some tinned salmon. One-half to three-quarters

* The preliminary articles of this Clinic appeared in The Hospital for August 19 and 26.

of an hour after partaking of the salmon all of them were seized with diarrhœa and vomiting and suffered from severe collapse. The woman, who was pregnant, aborted. All the patients recovered. The salmon is said to have had an " irony " taste which was so unpleasant that very little of it was eaten. The tin was sent to my laboratory, but except for a few flakes of salmon adhering to the edges it was empty. A number of these flakes were, however, examined, and from each of them an organism absolutely indistinguishable from the *pneumococcus* was isolated in pure culture. Three other tins were bought at the same shop and the contents of all proved to be sterile.

A few epidemics of food-poisoning have been recorded in which organisms other than those mentioned above have been believed to be the cause. But enough has been said to show that while in the great majority of cases of food-poisoning the disease is due to a specific infection with organisms of the enteritis group, symptoms of gastro-enteritis may nevertheless follow the consumption of food infected with one or other of several organisms belonging to other groups.

